# Elastocapillary effects determine early matrix deformation by glioblastoma cell spheroids

**DOI:** 10.1063/5.0191765

**Published:** 2024-05-03

**Authors:** Ida Ang, Muhammad Sulaiman Yousafzai, Vikrant Yadav, Kyle Mohler, Jesse Rinehart, Nikolaos Bouklas, Michael Murrell

**Affiliations:** 1 Department of Mechanical and Aerospace Engineering, Cornell University, Ithaca, New York 14853, USA; 2 Department of Biomedical Engineering, Yale University, New Haven, Connecticut 06520, USA; 3 Systems Biology Institute, Yale University, West Haven, Connecticut 06516, USA; 4 Department of Cellular and Molecular Physiology, Yale University, New Haven, Connecticut 06520, USA; 5 Center for Applied Mathematics, Cornell University, Ithaca, New York 14853, USA; 6 Department of Physics, Yale University, New Haven, Connecticut 06520, USA

## Abstract

During cancer pathogenesis, cell-generated mechanical stresses lead to dramatic alterations in the mechanical and organizational properties of the extracellular matrix (ECM). To date, contraction of the ECM is largely attributed to local mechanical stresses generated during cell invasion, but the impact of “elastocapillary” effects from surface tension on the tumor periphery has not been examined. Here, we embed glioblastoma cell spheroids within collagen gels, as a model of tumors within the ECM. We then modulate the surface tension of the spheroids, such that the spheroid contracts or expands. Surprisingly, in both cases, at the far-field, the ECM is contracted toward the spheroids prior to cellular migration from the spheroid into the ECM. Through computational simulation, we demonstrate that contraction of the ECM arises from a balance of spheroid surface tension, cell–ECM interactions, and time-dependent, poroelastic effects of the gel. This leads to the accumulation of ECM near the periphery of the spheroid and the contraction of the ECM without regard to the expansion or contraction of the spheroid. These results highlight the role of tissue-level surface stresses and fluid flow within the ECM in the regulation of cell–ECM interactions.

## I. INTRODUCTION

Coupled chemical and mechanical changes in the cancer micro-environment occur on diverse length scales, and facilitate the formation, growth, and metastasis of tumors.^1–4^ At the cellular level, cytoskeleton-induced mechanical forces drive morphological shape changes and migration.^5^ Cells also regulate osmotic pressure, cell volume, and fluid transport by ion channel and co-transporter activity.^6,7^ At larger length scales, the surrounding extracellular matrix (ECM) accumulates at the surface of the tumor. In collagen-rich ECM, at the perimeter of the tumor, the fibers are predominantly parallel to the surface and transition to a radial alignment further away from the surface. Ultimately, guided by this radial alignment, cells invade the ECM.^3,8^ However, in cell spheroids, commonly used as models of tumor invasion into the ECM,^9,10^ deformation of the ECM has been observed prior to the outward migration of cells.^11–13^ Furthermore, the magnitude of stresses generated at the spheroid periphery may be comparable to the bulk elasticity of both spheroids and the ECM on cellular length scales.^14–16^ This length, called the “elastocapillary length scale,” is the ratio of the surface energy (*γ*) to the initial shear modulus (*G*_0_), $l=\gamma /G_{0}$.^17^ Elastocapillary effects are typically negligible in solids on the macro-scale, but are an important consideration for soft materials at small length scales.^2,17–21^ Thus, tissue-scale elastocapillary effects may play a role in ECM deformation and ultimately impact cell invasion. However, the role of interfacial tension on the time-dependent properties of the ECM and eventual cell migration is poorly understood.

To date, the differential adhesion hypothesis (DAH)^22–24^ is a framework that considers interfacial energies that emerge at the macroscopic level due to the difference in cohesion between cells.^25,26^ In this case, differences in adhesion energies determine the sorting of mixed cell populations. As self-interactions are preferable to non-self-interactions, cell populations separate and envelop each other. In contrast, the differential interfacial tension hypothesis (DITH) includes active stresses in addition to adhesion.^27^ However, DAH and DITH are unable to describe the relationship between interfacial energy/tension and the deformation of soft solids, which evolves over time due to viscoelastic and poroelastic effects. Therefore, these hypotheses are not sufficient to fully describe the complex interactions between tumors and the micro-environment.

**FIG. 1.**
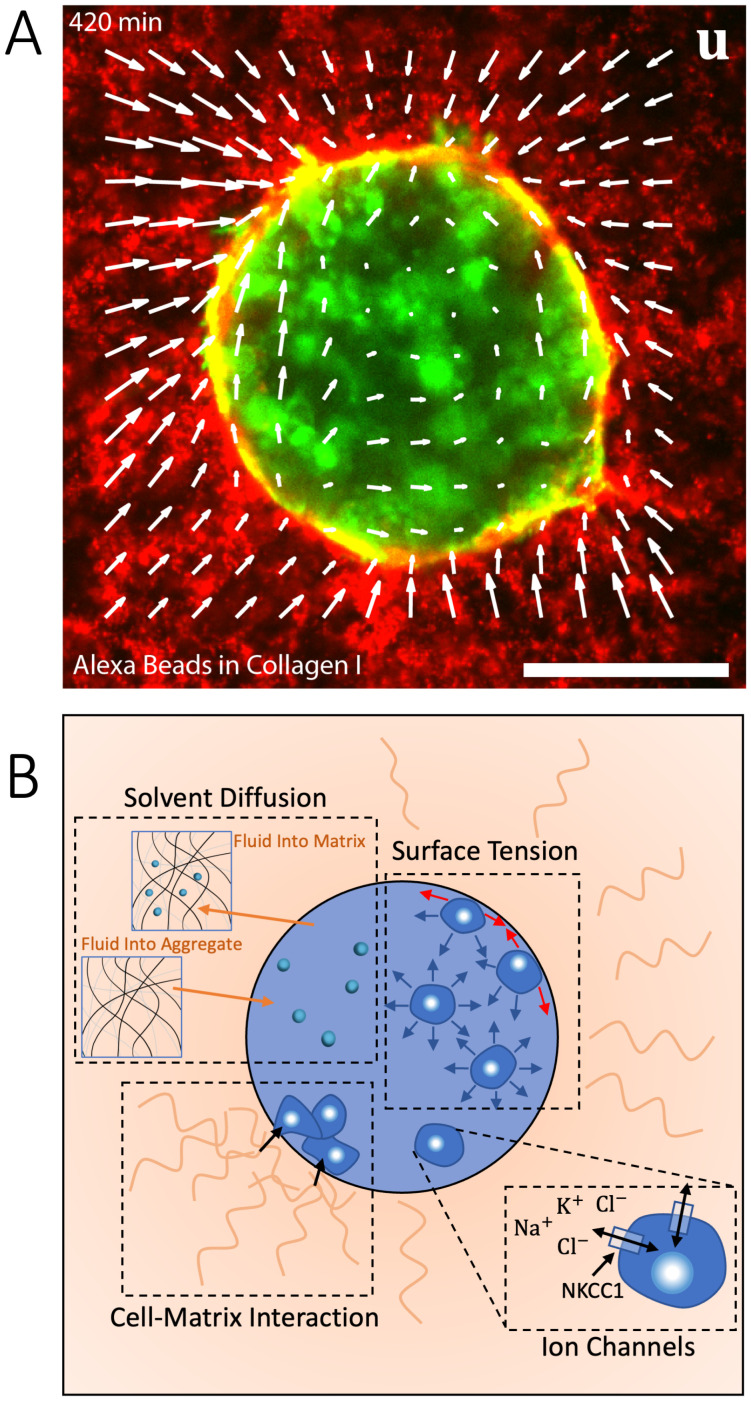
(a) Actin within GL261 cell spheroid (green) suspended in 1 mg/ml collagen I gel. The gel is labeled by the embedding of 100 nm Alexa Fluor beads (red). The spheroid contracts the gel, leading to a displacement, **u** (white arrow). Scale bar is 50 *μ*m. (b) Schematic of potential mechanisms from the cellular to the spheroid length scale which can induce overall deformation modalities. Mechanisms illustrated include (1) ion channel activity, (2) elastocapillary effects on cell spheroid from surface or interfacial tension, (3) solvent transport, and (4) and cell–matrix interaction. The sodium–potassium–chloride co-transporter 1 (NKCC1) channel on the cell membrane transports sodium, potassium, and two chloride ions, thus contributing to solvent diffusion as solvent migrates from areas of low- to high-ion concentration. Surface tension arises from a force imbalance between cells on the surface of the spheroid, while cells in the bulk experience an overall zero net force. Cell–matrix interaction arises from cells on the spheroid surface binding to collagen I within the extracellular matrix.

In this work, we explore four key mechanisms related to the mechanics of the spheroid/ECM interface through a combination of experiments and theoretical developments (Fig. 1). On the cellular level, (1) ion channel/co-transporter activity can influence solvent diffusion in and out of the cells, thereby impacting cell pressure and volume. As a result, cells in the bulk of the tumor experience forces from the surrounding cells, but for the peripheral cells, (2) surface (or interfacial) tension emerges macroscopically to maintain equilibrium due to a force imbalance between the peripheral cells and the spheroid interior. (3) Solvent transport between the ECM and spheroid allows accommodation of apparent swelling or compression in either phase, as all other constituents of both phases can be assumed to be nearly incompressible. Finally, (4) cell–ECM interactions have been shown to influence local ECM organization,^3,8^ where previous studies have demonstrated how the peripheral cells of the spheroid influence collagen fibrillar alignment.^11^ We, therefore, manipulate these properties in a model system, in which cancer cell spheroids are encapsulated in an ECM mimic [Fig. 1(a)].

Glioblastoma (GBM) is an aggressive form of cancer affecting the glial cells of the brain and spinal cord.^28,29^ Without treatment, life expectancy post-diagnosis is limited to within three months,^30^ due to the invasive potential of tumor cells and to tumor recurrence post-excision.^31^ Glioblastoma migration is driven by cytoskeleton-based protrusions and active stresses.^11,32^ Migration is facilitated by the coordination of ion channels and co-transporters,^33^ which cause large changes in cell volume.^34,35^ In GBM spheroids, the combination of these activities lead to large, internal capillary-driven deformations, suggestive of strong spheroid surface tensions (Fig. S1). Thus, due to the presence of capillary forces and potential for large changes in volume,^35^ GBM is a suitable model system for probing the role of spheroid surface tension and solvent transport on the deformation of the ECM.

The brain ECM includes fibronectin, laminins, tenascin, and glycosaminoglycans, such as hyaluronic acid.^36^ Collagen I is limited in gliomal tissue, although gliomal cells may produce their own.^37–39^ Thus, collagen I gels have been frequently used to study the invasion of gliomal cells.^40–48^ Glioblastoma cells bind readily to collagen and migrate within the matrix.^49^ Furthermore, the mechanical properties of collagen gels are similar to those of the brain ECM.^50,51^ Thus, collagen I gels are appropriate for demonstrating the role of interfacial tensions in deforming the ECM. We, therefore, utilize GBM cell spheroids embedded within collagen I gels, where cytoskeletal and ion co-transporter activity control the mechanical properties of the cell. In particular, as the latter has greater impacts on cell volume than the former,^52^ we focus on regulating ion co-transporter activity.

GBM cells regulate cell volume via the SPS1-related proline/alanine-rich kinase (SPAK) pathway through a direct phosphorylation of ion co-transporters that regulate migration-dependent cell volume changes and coupled cytoskeletal regulation.^35,53–55^ The physiological significance of this regulatory network had been confirmed by small molecules that target the NKCC1 and the central regulatory kinase SPAK.^56^ These studies establish that acute inhibition of ion transport impairs essential regulatory cell volume changes that impair a GBM cells' ability to migrate *in vitro* and *in vivo*. Furthermore, the regulatory response of the structural components of the cell and the extracellular environment have been investigated. Upon NKCC1 inhibition, the activity of signaling pathways (RhoA and Rac1) decreased, F-actin assembly is impaired, and cell spreading and migration decreases.^54^ Similar changes would be anticipated under SPAK inhibition where there are significant changes in cell volume. However, how cell volume, interfacial stresses, and ECM contractility are coupled remains unclear. In this study, we inhibit SPAK activity to induce cell volume change by impacting fluid diffusion across the cell. Modulating SPAK can lead to a decrease or increase in cell spheroid volume, which in turn exerts either tensile or compressive stresses on the ECM. Using GBM within collagen gels as a model system, we seek to explain the observation of ECM contractility without regard to the direction of volume change. In doing so, we suggest a phenomenon which depends only upon biophysical parameters of the tissue (spheroid) and the ECM, and, therefore, may be applicable to other systems composed of different cells or matrix components.

We analyze the deformation of the collagen gel subject to changes in the bulk mechanical properties of the spheroids. As the deformation patterns of the spheroid–ECM system are difficult to interpret due to the highly nonlinear coupling between force generation and deformations, a computational framework based on a continuum model and corresponding finite element (FE) formulation was developed. This model [Fig. 1(b)] considers (1) solvent diffusion within and between the cell spheroid and the ECM, coupled with the mechanics of each phase, (2) elastocapillary effects due to cell–cell adhesion on the cell spheroid periphery, (3) a zone for cell–ECM contractile interactions, and (4) modulation of the effective osmotic pressure within the cell spheroid due to ion channel regulation. This model can provide insight into the multi-physical effects occurring in the biological system isolating the effect of individual and pair-wise contributions. The FE formulation is motivated from a nonlinear poroelastic framework with surface effects which was developed to study drug delivery from hydrogel microspheres that exhibit surface effects.^57^ This numerical formulation has previously been extended to study contractile microtissues,^58^ where an active material model was introduced to allow for bulk contraction as well as surface contraction. We consider a fluid-like surface energy, where the surface free energy is constant per unit current area for consistency with the experimental measurements and leads to a constant surface Cauchy stress equivalent to surface tension. State-of-the-art computational models^59,60^ often focus on tumor growth, which is not the case here, as the focus is on the detailed interactions and transitions that lead to a response dominated from cell–cell effects to cell–ECM effects.

Two sets of experiments are conducted to examine (1) the cell spheroid deformation behavior by micropipette with and without SPAK inhibition and (2) the deformation of spheroids with and without SPAK inhibition suspended within a 1 mg/ml collagen gel. Furthermore, micropipette aspiration experiments are carried out on free cell spheroids, to measure surface and bulk properties. Previous studies have suggested that for roughly 1 h, the collective contractility of spheroids embedded within ECM mimics reflect the initial mechanical properties of individual cells. At longer time scales, contractility is altered by mechanical feedback through the ECM.^13^ Indeed, after this time, the ECM itself stiffens significantly,^61^ suggesting large changes in the interfacial tension.^62,63^ Thus, we assume that the surface tension measured by micropipette aspiration on spheroids in suspension relates only to the interfacial tension of spheroids within collagen gels occurring at early times ( ≤4 h). This ensures the strain in the network is kept below 2%–3% on average, a strain level where the collagen networks have been shown to stiffen by bulk rheology.^64^ We acknowledge the strain may be greater within optical resolution adjacent to the spheroid. We further assume the principal effects of our SPAK inhibitor are on modulation of cell volume and that potential off-target effects do not interfere with co-transporter activity in its impacts on volume change. Together, the experiments and simulations assist in understanding the roles of ion channel-mediated volume changes, cell–ECM interactions, and surface effects arising from cell–cell adhesion toward the eventual deformation of the cell spheroid and matrix system.

## II. RESULTS

### A. YU252 alters elastocapillary effects of cell spheroids by regulating cell–cell contact and volume

**FIG. 2.**
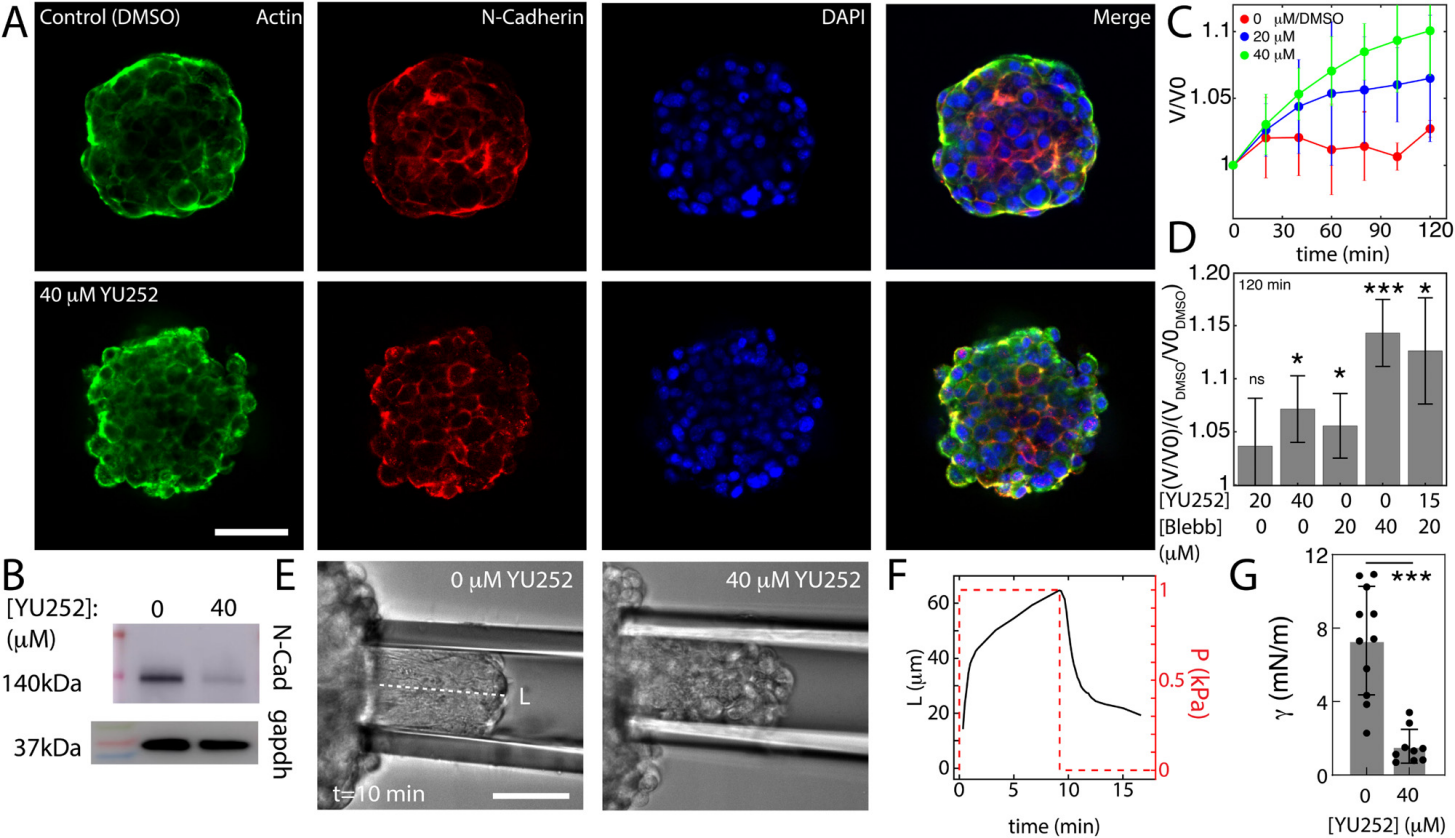
NKCC1 inhibition alters cell spheroid volume and mechanics. (a) Cellular spheroid stained to show actin, N-cadherin, and nuclear staining in untreated spheroid and spheroid treatment with 40 *μ*M YU252. Scale bar is 100 *μ*m. (b) Decrease in N-cadherin expression after YU252 treatment. (c) Normalized volume change $V(t)/V_{0}$ as a function of time for different concentrations of YU252. *V_DMSO_* reflects the volume of the control, in which DMSO is added. (d) Volume change relative to control at 120 min, comparing YU252 to blebbistatin treatment (n: 20 *μ*M YU252: 6, 40 *μ*M YU252: 4, 20 *μ*M Bleb: 5, 40 *μ*M Bleb: 5, 15 YU252/20 *μ*M Bleb: 5). ^*^ indicates p < 0.05. (e) Micropipette aspiration experiments on 0 *μ*M YU252 and 40 *μ*M YU252-treated spheroids. Spheroid is pulled into the pipette at a distance of *L*. Scale bar is 30 *μ*m. (f) *L* over time is subjected to the 1 kPa applied pressure. Rates of increase and decrease yield mechanical properties. (g) Surface tension, *γ*, decreases in magnitude on treatment with 40 *μ*M YU252 (n = 10, 9, N = 2). ^*^ indicates p < 0.05. ^***^ indicates p < 0.001.

YU252 is a potent pharmacological inhibitor of the SPAK pathway, with impacts on the sodium-potassium chloride transporter, NKCC1,^56^ known to play an active role in gliomal cancer progression.^35,53–55^ At the level of single cells, YU252 increases cell surface tension through a coordination of cytoskeleton and ion co-transporter-mediated forces.^7^ Cell volume increases, with concomitant changes in F-actin morphology and a decrease in motility. At the level of spheroids, the volume also increases. Here, we characterize the initial swelling of the spheroids, which ultimately leads to altered cadherin-mediated cell–cell contacts and a roughening of the surface of the spheroid (Fig. 2). We utilize YU252, as it creates changes in both cytoskeletal and membrane tension, without the same osmotic impacts on the collagen network, such as bundling or swelling that come from the addition of osmotically active agents.^65^

After formation by suspension spinning, spheroids in solution are stained for actin and N-cadherin [Fig. 2(a)]. Spheroids are then compared, under control conditions (DMSO) and 40 *μ*M YU252. Under both conditions, spheroids have peripherally, surface-localized actin as demonstrated by actin fluorescent intensity. Peripherally located F-actin is associated with elevated cell-generated stresses at the boundary of the spheroid compared to the interior of the spheroid.^14^ Localization of N-cadherin to the periphery suggests that cell–cell junctions are reinforced between cells at the surface compared to the bulk, which also suggests higher cell–cell derived forces at the boundary.^66–68^ In contrast, upon treatment with 40 *μ*M YU252, there is inconsistent quantities of actin along the spheroid surface and an overall reduction in the levels of N-cadherin consistent with previous reports in which NKCC1 is inhibited.^69^ Likewise, its peripheral localization also diminishes [Fig. 2(a)]. The decrease in N-cadherin is further confirmed through western blot [Fig. 2(b), Fig. S2]. Furthermore, other qualitative changes suggest a decrease in accumulated mechanical stress, such as a decrease in the circularity of the spheroid, and an increase in the circularity of the cell nuclei. The former suggests a decrease in the surface tension of the spheroid, and the latter suggests a decrease in mechanical stress applied to the cells, as the shape of nuclei reflects the stresses applied to the cells.^70^

In Fig. 2(c), the normalized volume change of spheroids as a function of time is plotted, demonstrating how treatment with YU252 leads to swelling of the cell spheroid. The spheroid volume increases significantly compared to untreated (DMSO). Interestingly, the increase in swelling has a similar effect as does inhibition of myosin II-based contractility [Fig. 2(d)], suggesting that the reduction in N-cadherin prevents the accumulation of cytoskeleton-mediated stress. Thus, there are numerous morphological changes and biochemical changes that indicate significant differences in the mechanical properties of cell spheroids upon treatment with YU252. We, therefore, measure the mechanical properties of spheroids with micropipette aspiration.

Micropipette experiments are performed on spheroids after 3 h of treatment to measure surface tension [Fig. 2(e)]. At this point, the change in volume approaches a maximum value. For the control condition (DMSO, 0 *μ*M YU252 treatment), the spheroid enters the pipette at a distance *L*, and the cells are elongated, reflective of the applied mechanical stress. In contrast, with 40 *μ*M YU252 treatment, the cells are less elongated as they enter the pipette.^71,72^ Furthermore, the rates at which *L* increases and decreases when subject to the applied pressure is used to estimate the surface tension of the spheroid [Fig. 2(f)]. As displayed in Fig. 2(g), the surface tension (*γ*) for control spheroids is 7.2 ± 3 mN/m (N = 10), while 40 *μ*M YU252 treatment results in a decrease in surface tension of 1.3 ± 1 mN/m (N = 9), as well as a decrease in the viscosity and elasticity (Fig. S3). The decrease in surface tension is consistent with the qualitative changes in N-cadherin,^64,73^ as well as spheroid and nuclear shape.

### B. Role of cell–cell and cell–matrix interactions in ECM deformation modalities

**FIG. 3.**
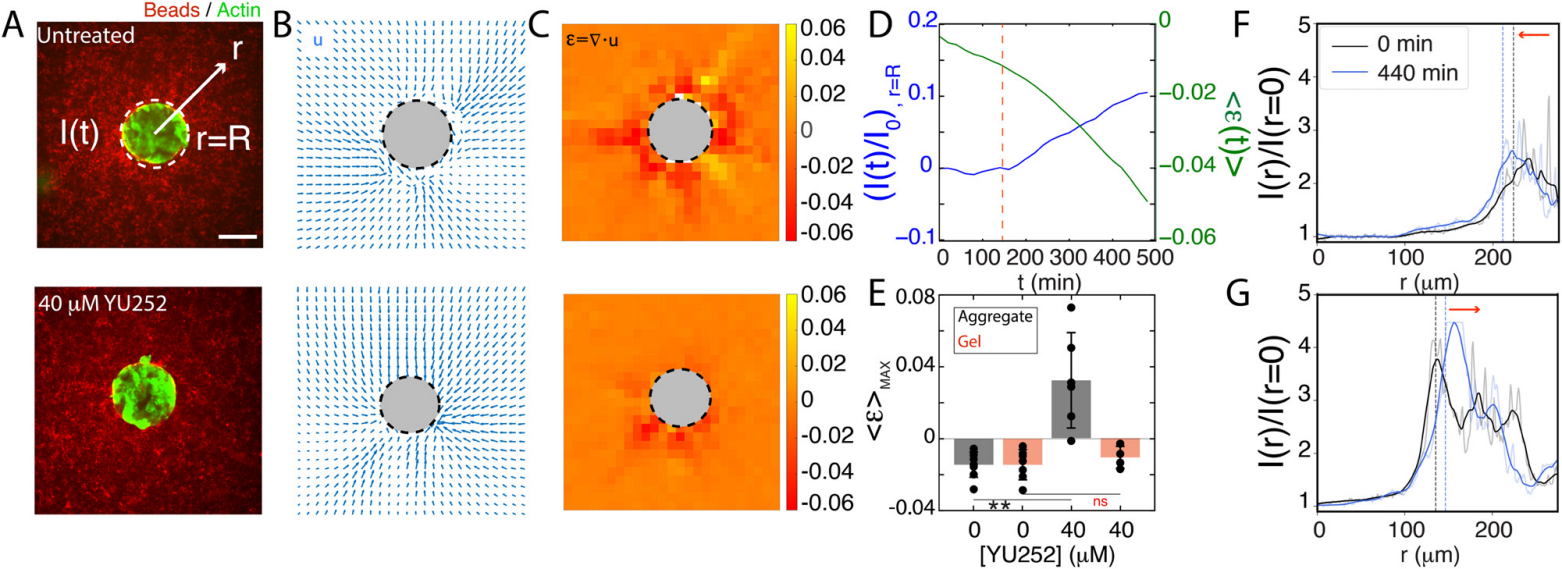
ECM is accumulated at spheroid surface during expansion. (a) Representative fluorescence images of spheroids (green) within collagen matrix (red). White dotted line indicates fluorescence of beads at the spheroid surface (*r* = *R*), *I*(*t*). (b) Displacement field ( $\vec{u}$), with spheroid excluded (circle). (c) Volumetric component of the small deformation strain tensor, *ε*. Colormap indicates magnitude of *ε*. (d) Measurement of *ε*, averaged over the field of view ⟨ε⟩, plotted with the change in fluorescence intensity of the beads at the surface [*I*(*t*)] compared to its initial value (*I*_0_). (e) Mean *ε* in spheroid and gel for control and YU252-treated cases, measured at 120 min. ^**^ implies P < 0.01, ns is non-significant (DMSO control: n = 7/7, YU252-treated: *n* = 6/4, *N* = 2). Profiles of normalized intensity(to the center of the spheroid) measured for representative examples of the (f) control and (g) YU252-treated cases. Raw and filtered profile data are displayed with transparent and opaque lines, respectively. Data are filtered with a Savitzky–Golay filter with a window length of 20 and a polynomial order of one. The dashed vertical lines demonstrate the radius of the cell spheroid decreasing from 223 to 211 *μ*m for the control and increasing from 135 to 146 *μ*m for the treated cases. Fluorescence image insets demonstrate the embedded gel (labeled red beads) at time 0 and 440 min. All scale bars are 100 *μ*m. ^**^ indicates p < 0.01.

Spheroids are suspended within 1 mg/ml collagen I gels, where the gel mimics the tumor ECM and maintains the spheroids in suspension [Fig. 3(a)]. Prior to collagen gelation, 200 nm Alexa Fluor beads (Molecular Probes) are mixed within the collagen and remain suspended during the experiment. In the case of YU252 treatment, the gels have been polymerized in the presence of the compound, and, thus, the spheroids are first exposed to YU252 upon being added to the gel (t = 0 min). After addition of the spheroids, their shape, the motion of cells within them, as well as the motion of the collagen are measured over time. Thus, here we select for comparison spheroids that contract and ones that expand to understand how the bulk and surface properties of spheroids impact the mechanical behavior of the spheroid-ECM system.

The motion of the collagen is visualized by brightfield as well as the fluorescence of Alexa Fluor beads, as demonstrated in Fig. 3(b). In both cases, the displacement is measured by particle image velocimetry (PIV), through the accumulated displacement field, **u**, which can be used to measure the volumetric (scalar) component of the (infinitesimal) strain tensor in the collagen network, denoted by ε=∇·u [Fig. 3(c)]. From the spatial pattern of the strain field, in the control sample, we observe high contractility immediately proximal to the spheroid surface, which then decays away from the surface. In contrast, upon YU252 treatment, consistent with the decrease in spheroid surface tension, there is also a decrease in the magnitude of the strain (Fig. S4).

The motion and morphology of the spheroids are also monitored over time. When control (DMSO) cell spheroids without treatment are placed within the collagen gels, the overall cross-sectional area of the spheroid decreases, suggesting a modest decrease in the volume of the spheroid [Fig. 3(e)]. Here, this is measured as a negative volumetric strain, in plane strain conditions. This negative volumetric strain is consistent with a negative volumetric strain in the surrounding gel [Fig. 3(d)]. In contrast, as shown earlier, there is an increase in the volume of the spheroid when treated by YU252. Surprisingly, there is still a negative volumetric strain in the surrounding gel. Despite the overall average negative volumetric strain within the gel, at the surface of the gel, the mean fluorescence intensity of the beads either moves inward for control spheroids [Fig. 3(f)] or outwards for YU252-treated spheroids [Fig. 3(g)]. For the latter, it can be seen that an increase in fluorescence intensity *I*(*t*) at the spheroid surface is consistent with the decrease in strain, suggesting the two are coupled. Contraction of the spheroid in the control suggests that interfacial (surface) tension minimizes spheroid surface area. Likewise, in the YU252-treated case, the expansion of the spheroid reflects a decrease in that interfacial tension. Furthermore, the movement of the collagen gel inward as the cell spheroid expands suggests low or negligible tension and another mechanism beyond fluid diffusion at play. In the case of the YU252-treated samples, there is a seeming incompatibility of the motion of the spheroid (outward motion) and the gel (inward motion) that up to this point remains unresolved.

The accumulation of bead fluorescent markers at the surface of the spheroid suggests a cell–matrix interaction that has been independently postulated in other experimental work.^11^ In this case, cells on the outer layer of the spheroid stretch and protrude into the matrix^74^ (Fig. S5). The precise mechanism for ECM accumulation is unclear. However, it is likely attributable to accumulation due to spheroid expansion as well as inward, cell-generated traction forces. In either case, this cell–matrix adhesion activity is distinct from surface tension (which is a result of cell–cell adhesions), and, therefore, indicates an additional mechanism that reconciles the apparent incompatibility of the motion of the treated spheroid and surrounding gel.

### C. Simulations

Our theoretical framework is based on prior nonlinear poroelastic theories^75–77^ that consider the effects of solvent diffusion and large deformations. We consider both the gel and spheroid as poroelastic media with distinct properties, where the hydrogel constituents are assumed to be incompressible^75,78^ and hydrogel volume changes only through fluid diffusion. Effectively, the osmotic pressure is modulated in the spheroid portion of the poroelastic medium, to capture the action of ion channels. To consider cell-derived surface tension in the context of large deformation poroelasticity and develop a finite element transient solution strategy, we follow our previous work.^57,58^

The goal of the FE simulations is to lend intuition to the complex multi-physical processes occurring in the experimental setup at multiple length scales and time scales. To balance formulation complexity with geometric simplicity, we consider a 2D domain in plane strain conditions, and distinguish between an exterior region which represents the gel/matrix and an interior circular region which represents the cell spheroid.

**FIG. 4.**
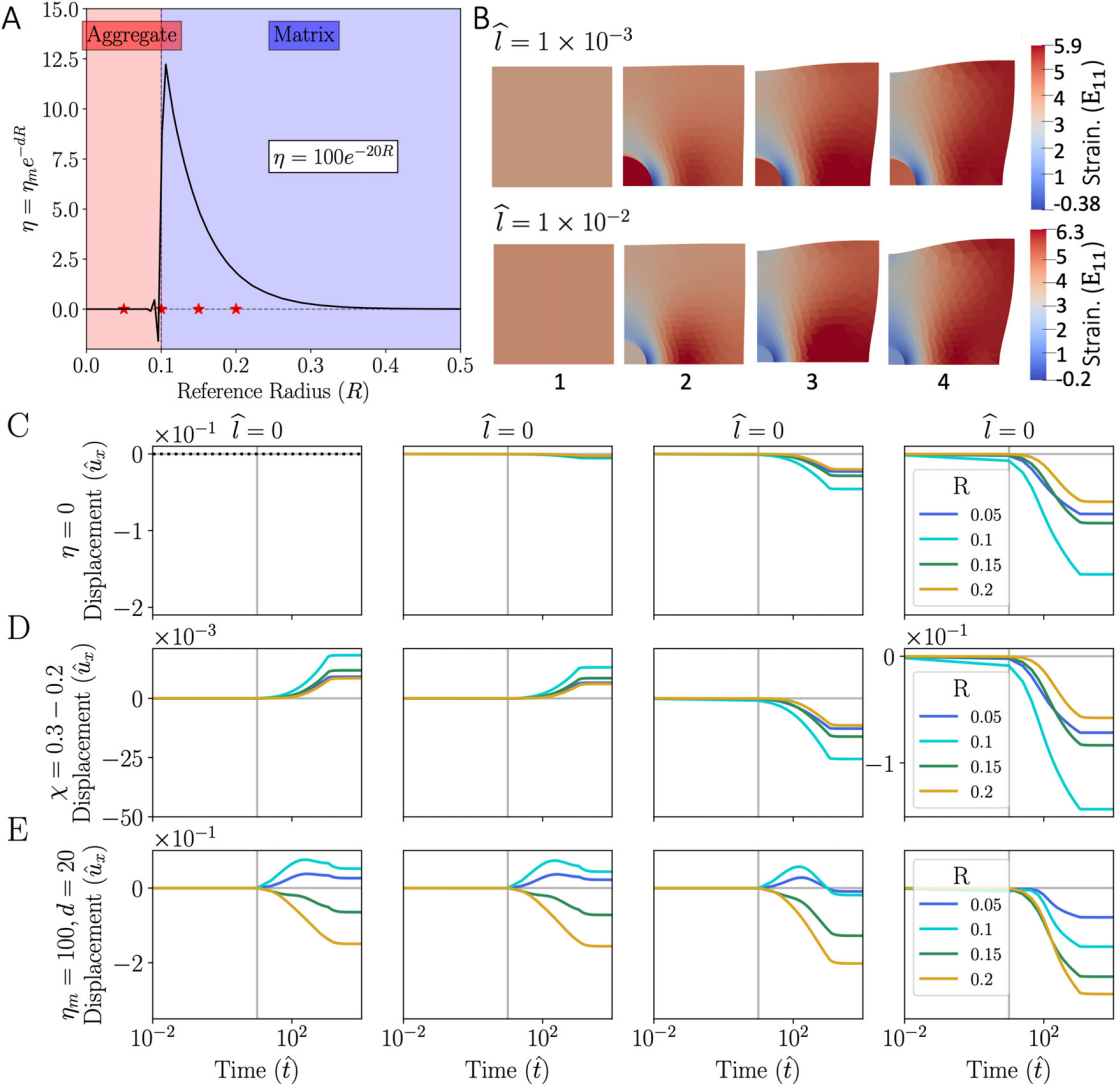
Simulation setup and results. (a) The interface between the “spheroid” and the “extracellular matrix” is outlined and points from which displacement is tracked at $X_{1}=0.05,\;0.1,\;0.15,\;\text{and}\;0.2$ are displayed with red stars. The decaying exponential function $\eta =\eta_{m}e-(dR)$ defines a bulk contractility constant where $\eta_{m}=100\;\text{and}\;d=20$. (b) Four deformed configuration instances of the *E*_11_ component of Green strain at $t=2\times 10^{-3},\;3.13\times 10^{2},\;1.1\times 10^{3},\;\text{and}\;7.1\times 10^{3}$ for two cases with varying levels of surface tension applied in the presence of cell–matrix interactions and cells collecting on the periphery of the spheroid. (c) Displacement at four points over time where only surface tension is applied on the spheroid. (d) Displacements where Flory parameter, *χ*, is adjusted from 0.3 to 0.2 within the cell spheroid domain, with and without different magnitudes of surface tension. (e) Displacements for a case demonstrating just the effects of an active contractile force defined with a decaying exponential function, and cases with combined effects of contractile force and surface tension ( $\hat{l}=1\times 10^{-3},\;1\times 10^{-2},\;\text{and}\;1\times 10^{-1}$).

Following the theory set forth in Bouklas *et al.*,^77^ the baseline for both phases of the poroelastic model pertaining to the results in Fig. 4 is $N_{e}\Omega =10^{-3}$ and an interaction (Flory) parameter of χ=0.3, which describes the enthalpic interaction of the solvent and polymer. Three additional effects can be incorporated in the model to study their influence on the eventual outcome. First, the interface of the cell spheroid, which is modeled with a circle of radius *R* = 0.1 in the reference configuration, can have a constant surface free energy *γ* per unit reference area. This surface free energy, *γ*, induces a constant surface tension in the deformed configuration mimicking potential elastocapillary effects from cell–cell adhesion. The surface tension can be allowed to increase due to the stabilization of the cell–cell adhesions, which in the simulation is facilitated through ramping of *γ* [Eq. (S24)]. Second, the Flory parameter in the spheroid can be adjusted to model a more hydrophilic cell spheroid, allowing for the study of the hypothesis that ion channel activation can increase osmotic pressure in the spheroid accommodated by fluid transport. Finally, the cell–matrix interaction on the spheroid periphery is modeled through a contractile body force that is acting on a near-surface region defined by the exponential decay function, $\eta =\eta_{m}e^{-d\sqrt{X_{1}^{2}+X_{2}^{2}}}=\eta_{m}e^{-dR}$ [visualized in Fig. 4(a)], which can be controlled to adjust a level of cell-induced ECM contraction. Notably, these various effects can be examined independently, and then combined to develop intuition about the experimental system. It is also important to note that the cell–ECM interactions are not restricted to an interface but actually a finite volume (i.e., a hollow thick shell) on the exterior of the spheroid. In contrast, in the differential adhesion hypothesis, cell–ECM interactions are only taken into account through an interfacial contribution.

The effects of surface tension and cell–matrix interaction are first modeled separately and then combined. Figure 4(a) displays the exponential decay function used to model matrix contraction and the four points used to track displacement of the spheroid, interface, and matrix. First, we can examine the effects of just surface tension for the cell spheroid. The elastocapillary length scale is defined as $l=\gamma /G_{0}$, the ratio of the surface tension to the instantaneous/initial shear modulus. The magnitude of the surface tension is linearly increased from zero to its final value reminiscent of stabilization of cell–cell adhesion. Surface energy can dominate the response when the elastocapillary length scale $\hat{l}$ is larger than the diameter of the spheroid as seen in Fig. 4(c). Increasing the magnitude of surface tension induces an inward transient motion for the spheroid and the matrix, which is accommodated in the poroelastic medium by the corresponding fluid transport. Surface tension induces a response reminiscent of the control. Normalizations are indicated with a hat, where $\hat{l}$ indicates the normalized elastocapillary length scale and $\hat{t}$ the normalized time.

Next, ion channel-mediated fluid diffusion is modeled by decreasing the Flory parameter within the interior of the domain (the region that represents the cell spheroid) from χ=0.3 to χ=0.2. By changing the interaction parameter to a lower value, the spheroid swells. Notably, the system moves together in either expansion or contraction as we modulate the level of surface tension as seen in Fig. 4(d), and does not exhibit a difference in behavior between the cell spheroid and the matrix (all inward or all outward motion). This result leads to the observation that swelling driven by osmotic pressure changes cannot single-handedly induce the complex response of the treated system (inward motion for matrix, outward for gel).

Finally, to incorporate the effect of cell–matrix interactions, the exponential decay function is utilized in the poroelastic model, with parameters $\eta_{m}=100\;\text{and}\;d=20$. In the absence of surface tension, or at low surface tension ( $\hat{l}<10^{-2}$) incorporating the effect of cell–matrix interactions leads to an outward radial motion for the spheroid and an inward radial motion for the matrix [seen in Fig. 4(e)], reminiscent of the response of the treated samples. It has to be noted that the cells collecting on the periphery of the spheroid are highly compressed in the radial direction as seen in Fig. 4(b) even for higher levels of surface tension. Application of higher surface tension, $\hat{l}>=1\times 10^{-2}$, changes the modality of the deformation, where the equilibrium deformation indicates a radially inward motion for both the spheroid and the matrix [Fig. 4(e)].

Figure 4(b) shows the strain measure **E**. The Green strain tensor is defined as follows: $E=\frac{1}{2}(F^{T}F-I)$, where F=I−∇u is the deformation gradient and **I** is the identity tensor. The instances of strain in Fig. 4(b), *E*_11_, correspond to the displacements in Fig. 4(e).

## III. DISCUSSION

To date, contraction of the ECM is attributed principally to the accumulation of tensile stresses due to cellular level contractility during invasion.^79^ Adhesion–contraction coupling, a mechanism by which mature focal adhesions couple to strong actomyosin-based mechanical forces, guides outward motion by inducing alignment of the extracellular matrix.^80–84^ In contrast, other works have suggested that the bulk contraction of tissues themselves can generate forces prior to the activity of individual cells.^11–13^ Furthermore, some works have reported expansion of the gel, although typically by cells that have ultimately separated from the spheroid,^12^ or relaxations of contractile stresses generated by cells invading outward.^13^

Here, we explain our principal observation, the contraction of the ECM given the contraction or expansion of a cell spheroid. Previous studies have indicated on small scales, that “rectification” and net contractility of biopolymer networks is a result of the mechanical properties of the network itself.^85,86^ Our results suggest that inward radial motion of the matrix is a consequence of collective cell–cell adhesion and manifestation of surface effects and is not exclusively generated by individual cells directly interacting with the matrix (on the contrary, that would lead to a negative pressure and expansion of the spheroid). This assumption is enforced by the results from Fig. 2(a): Control, where the cell spheroid without the presence of the matrix minimizes the surface area and attains a spherical shape, while at the same time expelling the solvent. This result can be attributed to surface tension alone. Disruption of surface tension by YU252 treatment ultimately leads to a loss in overall spheroid circularity [Fig. 2(a)] and a significant reduction of surface tension on the cell spheroid surface[ Fig. 2(d)].

Upon placement in the collagen matrix, control cell spheroids adhere and induce an inward radial motion to the matrix [Fig. 3(a)]. The volume of the spheroid decreases, with the spheroid being in a state of hydrostatic compression induced by the Laplace pressure due to surface tension. Contraction upon placement of the spheroid in the gel can be understood as maturation and stabilization of cell–cell adhesion in the presence of ECM; the corresponding rise of surface tension during maturation, and ion channel activity, exerts a hydrostatic pressure to the spheroid driving the expelling of solvent and the corresponding contraction.

Inhibition of NKCC1 activity with YU252 treatment induces concurrent spheroid swelling accompanied by an apparent inward motion of the matrix. This result is surprising, and also seemingly incompatible, because the assumption is that as the cell spheroid swells due to an influx of water from the surrounding gel, the elastic matrix will deform and normal stresses on the collagen gel act radially outward leading to the gel moving outward as well. The simulation [Fig. 4(d): $\hat{l}=0$] demonstrates just fluid diffusion due to increased swelling of the spheroid due to ion channel activity, which leads to an overall displacement radially outward for both the matrix and the spheroid. The addition of higher surface stresses leads to an inversion of the response leading to both the spheroid and the matrix moving radially inward. Since osmotic swelling counteracts the Laplace pressure from surface tension, this observed result and inversion is reasonable. Therefore, considering swelling effects from an ion channel treatment along with surface tension from cell–cell adhesions cannot completely explain the YU252-treated results. Thus, the concurrent cell spheroid outward with matrix inward motion must be due to another mechanism.

Figure 3(f) and Fig. S5 demonstrate fluorescence markers collecting close to the spheroid surface, forming a layer, and generation of contractile strains around the spheroid periphery. This suggests that the ECM is concentrated at the spheroid surface as the spheroid expands, creating an ECM shell around the spheroid. Reorganization of collagen is reminiscent of tumor-associated collagen signatures (TACS).^81,87^ TACS I, reflects the dense collagen immediately parallel to the tumor, without clear large-scale alignment. TACS II reflects the less dense later proximal to TACS I, and TACS III reflects the well-known aligned collagen that has been shown to enhance migration.^88^ While the accumulation observed may be due to many factors, including *de novo* collagen synthesis, as well as penetration of the collagen into the first few layers of cells,^11^ which is not considered here, the outward motion of beads indicates physical forces that contribute to their accumulation. It is further a possibility that, with the local accumulation and compaction of collagen,^89^ there are impacts on cell proliferation^90^ or changes in cell spreading and contractile behavior^91^ due to changes in matrix rigidity, adhesivity, or porosity.^92^ This may serve to counteract the decrease in migration at the single cell level that occurs with YU252 treatment.^54^ Further analysis of the dynamics of collagen fibers during expansion and outward motion are needed to confirm the precise mechanism.

These experiments motivated the modeling of the layer of cells at the periphery of the spheroid with an exponential decay function which acts directly on a near-surface zone of the matrix resembling the action of cells to the matrix. The combined effect of cell–matrix interaction, surface tension, and solvent exchange in the simulation assists in explaining how both spheroid swelling and apparent ECM inward motion can occur simultaneously. The key is the consideration of the complex deformation gradients close to the spheroid surface, where cells interact with the surrounding ECM.

The simulation considering the layer of cells collecting at the periphery of the spheroid [Fig. 4(e) $\eta =100e^{-20R},\;\hat{l}=0$] recapitulates the response of the treated sample; namely, the matrix moves inward, the spheroid moves outward, and a highly compressed zone forms. Nevertheless, we cannot dismiss the possible effects of surface tension combined with cell–matrix interaction, since the rounding of the cell spheroid is observed at early times (with roughening at late times) as more fluid permeates the cell spheroid and spheroid volume increases. Figure 4(e) ( $\eta =100e^{-20R},\;\hat{l}=1\times 10^{-3}$) demonstrates the combined effects of surface tension and cell–ECM interaction which demonstrates a similar result to the experimental treatment of YU252. The strain snapshots in Fig. 4(b) shows how an order of magnitude difference in surface tension while maintaining the same level of cell–matrix interactions can lead to very different outcomes.

The main aim of the computational model and simulation is to enable hypothesis-driven research by allowing for detailed control of the mechanisms that control the response of the spheroid–ECM system. As such, this enables a qualitative understanding of the system constrained by the modeling assumptions; namely, the choice of (1) plane strain conditions, (2) the nonlinear poroelasticity model for the spheroid and the ECM, (3) fluid-like surface tension, (4) the choice of an isotropic model that does not consider fiber rearrangement for the ECM, and (5) the model for cell–ECM interaction. To accurately recapitulate the complex multi-physical processes occurring in the system at multiple length scales and time scales, some of these modeling choices can be further improved and validated by experiments.

In conclusion, surface forces generated from cell–cell adhesion can explain the results within the control through the manifestation of surface tension combined with poroelastic effects that allow for solvent transport coupled with large deformations. In contrast, elastocapillary effects alone, ion-mediated diffusion alone, and these two effects coupled together are not able to mimic the concurrent spheroid outward motion and ECM inward motion observed in the YU252 experimental results. Elastocapillary effects dominate in all cases when the elastocapillary length scale is equivalent to the radius of the spheroid. Therefore, it is important to consider the effects of cell–ECM interaction, which is reinforced by the experiments demonstrating fluorescent beads collecting on the surface of the cell spheroid. This interaction between the cells and collagen within the matrix, modeled with an exponential decay function, leads to simulation results that show a split in the behavior of the cell spheroid and the matrix by the formation of a radially compressed zone. Not only have we provided measurements for surface tension for cell spheroids, indicating its disruption by YU252 treatment but we have also followed the different deformation modalities in control and treated spheroids when placed in the matrix. The difference in deformation modalities was shown to be a direct consequence of surface tension due to the prevalence of cell–cell adhesion or due to the formation of a highly compressed zone in the periphery of the spheroid due to the prevalence of cell–matrix interaction consistent with the assumptions for the YU252 treatment.

While we have characterized and validated on-target activity for YU252 in this study, it is important to note that off-target activity cannot be ruled out without performing more in-depth cellular profiling. Parallel studies with an orthogonal, highly selective SPAK inhibitor could help to delineate off- and on-target activities. *In vitro* selectivity profiling is available in Ref. 56. While some off-target kinases may overlap with those controlled here, no off-target kinases phosphorylate NKCC1/KCC and control cell volume.

Mechanical force generation and the adhesive interactions that connect the cell to the ECM are targets for potential therapy against cancer metastasis.^93^ The results presented here may provide information on the engineering and design of artificial matrices that manipulate these processes. The inclusion of elastocapillary effects determines the extent to which tissue-scale stresses contribute to cell motion and remodeling of the ECM. Thus, the elastocapillary length, in addition to more well-understood properties, such as ECM rigidity, topology, adhesivity, and porosity,^94–98^ factor into the design of matrices that facilitate or inhibit motion. As cell spheroids are commonly used as simple models to screen cancer therapeutics or chemotherapy,^99–101^ there is a potential that invasion subject to intervention has separate outcomes depending upon the presence of elastocapillary effects.

## IV. METHODS

### A. Cell culture

Glioma GL261 mouse cells are cultured at 37 °C under 95% air/5% CO_2_ atmosphere in culture medium consisting of Dulbecco's modified eagle medium (DMEM) enriched with 10% calf serum and 5% penicillin streptomycin (Penstrep). Cell spheroids are prepared from confluent cell cultures using the suspension-spinning method. Spheroids ranging from 80 to 450 *μ*m in diameter are obtained from 5 ml of cell suspension in CO_2_-equilibrated culture medium at a concentration of $4\times 10^{5}$ cells per ml after placement in a gyratory orbital shaker at 75 rpm at 37 °C for 30–50 h. The flasks are pretreated with 2% dimethylchlorosilane in chloroform to prevent adhesion of cells to the glass surface.

### B. F-actin transfection

Glioma GL261 cells were stably transfected with plasmid construct encoding for F-TRActin-EGFP. Cells were transfected using FuGENE ® HD Transfection reagents where 2 *μ*g of the DNA plasmid was added to the transfection reagent, added to a cell dish, then incubated for over 24 h with the plasmid to complete the transfection process. Following 1 week of incubation with a selection media containing G418 (Mirus Bio LLC) at 1 mg/ml, the population of cells was isolated, flow sorted with FACS flow cytometer, cultured, expanded in DMEM (supplemented with 10% FBS, 1% Penstrep), and used for experiments.

### C. Immunofluorescence staining

Adherent and non-adherent cells were fixed with a solution of 4% paraformaldehyde in 1X PBS for 15 min and permeabilized with 0.2% Triton X-100 for 20 min. The cells were then blocked with 2% bovine serum albumin (BSA) in 1X PBS (PBS-2% BSA) at room temperature for 1 h, incubated with primary antibodies for paxillin (recombinant anti-paxillin antibody [Y113], Abcam ab32084; 1:250 dilution) and myosin [phospho-myosin light chain 2 (Ser19) antibody, Cell Signaling Technologies^®^ (# 3671); 1:250 dilution] in PBS-2% BSA for 48 h at 4 °C. The sample was incubated fo0r 48 h at 4 °C for secondary antibodies Alexa Fluor 647 (donkey anti-rabbit, Abcam ab 150075, 1:500 dilution) and Alexa Fluor 555 [anti-mouse IgG (H + L), F(ab ′)2 fragment, 1:500 dilution]. Phalloidin staining was performed with Alexa Fluor 488 phalloidin (Life Technologies; 1:200) diluted in PBS with 2% BSA for 48 h at 4°C. Images were taken with 2X (air), 40X, and 60X oil immersion objective. Analysis was performed with ImageJ and Imaris (Bitplane).

### D. Micropipette aspiration

Micropipette aspiration of the spheroids was performed at physiological temperatures of 37 °C using pipettes fabricated from borosilicate with diameters 3–5 times that of a single cell (40–70 *μ*m). Thus, we assume that the spheroid can still be treated as a continuum and, therefore, can be characterized by parameters such as surface tension, elasticity, and viscosity.^71^ Spheroids were then suspended in a CO_2_-equilibrated culture medium, and the pipette was introduced into the chamber. A range of pressures (500 Pa to 1.5 kPa) can be attained by vertically displacing a water reservoir connected to the pipette, with respect to the observation chamber (the microscopic slide). Aspirated spheroids were visualized on an inverted microscope equipped with a 20× air objective. Movies of the advancement of the spheroids inside the pipette were recorded with a CCD camera with a 5–30-s interval.

### E. Collagen gel preparation

To prepare the collagen gel, we mixed 4 mg/ml stock solution of collagen I with 0.1% W/V NaOH, 5X DMEM, and water. Cell spheroid solution (200 *μ*l) was added to a final concentration of 1 mg/ml of collagen, then the mix was polymerized in a Chamlide chamber for observation. For YU252-treated cases, YU252 was added during polymerization, where 40 *μ*m was for measurement of mechanics in solution and within 3D gels. For imaging the collagen, 0.2 *μ*m red fluorescent microspheres (molecular probes) were embedded within the gel during polymerization.^12^ The size of the beads is slightly less than the pore size measured at this concentration of collagen I.^102^ However, we see no indication of bead diffusivity, and sought to maintain a high density of beads for high-resolution visualization and calculation of ECM deformation. We also keep this concentration constant, as changes in pore size can lead to differences in migration.^103^ Furthermore, linear rheology of collagen gels yield elasticities on the order of 10–100 Pa,^104,105^ and nonlinear measurements can increase the elasticity to over 100–1000 Pa.^64,106^

### F. Laser ablation

We use a high-energy laser (*λ* = 337 nm, 40 mW) to ablate cells at a point along the surface of non-adherent GL261 cells (Fig. S1).^16^ As the laser is focused at a 0.6 *μ*m diameter area (20× objective, NA 0.8), one cell is immediately killed, and often a small number of surrounding cells also become necrotic.

### G. Statistical tests

All statistical comparisons between two distributions were done with a two-sided t-test. *N* is the number of independent experiments and n represents the total population size. When distributions are presented as a single value with error bars, the value is the mean of the distribution, and the error bars are the standard deviations. We use the symbols ^*^, ^**^, and ^***^ for p<0.05, 0.01, and 0.001, respectively. When fitting lines to data, we quote the p-value as significance values to rejections of the null hypothesis.

### H. Image analysis

Particle image velocimetry (PIV) is applied to fluorescent and brightfield images. The code is based on custom-written routines in MATLAB (Mathworks).

### I. Simulation setup

All simulations were implemented in FEniCS, which is an open-source finite element (FE) software for automated solutions to differential equations.^107,108^ A two-dimensional mesh (Gmsh v.4.4.1) was set up such that an interface between the “cell spheroid” of radius, *R* = 0.1, and the “extracellular matrix” is clearly defined. Horizontal and vertical centerlines from the interface to the boundary are defined to allow for roller displacement boundary conditions (i.e., for the horizontal and vertical centerline, displacement is constrained in the *X*_2_ and *X*_1_ direction, respectively).

As a time-dependent system, it is important to also define an initial chemical potential, *μ*_0_. The definition of *μ*_0_ is dependent on $N_{e},\;\Omega ,\;\lambda_{0},\;\text{and}\;\chi$, the effective number of polymer chains per unit volume of the polymer, the volume of a solvent molecule, the initial stretch of the system, and the Flory parameter. For more information, see supplementary material, Sec. II, E: Initial Conditions, Eq. (S23). We assume the initial state is a fully swollen state, where *λ*_0_ is defined by setting the initial chemical potential to zero ( $\mu_{0}=0$). For the simulations, the initial time step for ramping is set to Δt=10 allowing for a slow change in either the contractility constant or the surface energy applied for convergence purposes. Time-ramping, where the surface tension (*γ*) is increased, Flory parameter (*χ*) decreased, or the contractility constant (*η*) is increased, is fictitious and is adjusted according to convergence and when the equilibrium state is achieved.

## SUPPLEMENTARY MATERIAL

See the supplementary material for Sec. I, which contains a calculation of the elastocapillary length for spheroids, as well as the following five supplementary figures: Fig. S1: Surface tension gradients drive internal flows in GL261 spheroids. Upon ablation of cells at the surface, rapid surface and bulk motions are observed within the spheroids. This indicates that surface tension gradients created by ablation are significant enough to yield deformations,^16^ thereby motivating an exploration of their effect on the ECM. Inhibition of active stress by blebbistatin leaves spheroids round (suggesting the presence of passive stress), but inhibits all flow. Figure S2: N-cadherin expression is reduced in YU252-treated spheroids. Two western blots that indicate the reduction in N-cadherin expression in YU252 compared to control. Figure S3: Viscoelasticity of GL261 spheroids. The elasticity and viscosity of spheroids as measured by micropipette aspiration, under control conditions, and when treated with the SPAK inhibitor, YU252. This demonstrates that there is a reduction in both the viscosity and elasticity of the spheroids under YU252 treatment. This contrasts to the impact on single cells.^7^ Figure S4: Collagen gel deformation approximates the elastocapillary length. Prior to significant outward migration by cells, the ECM is deformed. The length scale of that deformation is in the order of the estimated elastocapillary length. Figure S5: Cell protrusions activity in 40 *μ*M YU252-treated aggregates. Cells migrate outwards after accumulating collagen at their periphery. As there is a reduction in traction stress and protrusion. as demonstrated previously at the single cell level,^54^ the enhancement of collagen and local stiffening may recover some migratory activity. Section II outlines the continuum theory, covering the kinematics, the equilibrium and constitutive relationships of a poroelastic system, the mixed finite element formulation which is implemented in *FEniCS*,^107,108^ and the initial conditions for the model.

## Data Availability

The data that support the findings of this study are available from the corresponding authors upon reasonable request.
